# Pain Evaluation after Pulsed Radiofrequency in Patients with Osteoarthritis of the Hip

**DOI:** 10.1055/s-0044-1800937

**Published:** 2025-06-23

**Authors:** Rafaela Reis Torrealba, Phercyles Veiga-Santos, Maria Isabella Cruz de Castro, Lourenço Peixoto, Marcelo Felipe Almeida, Conrado Torres Laett

**Affiliations:** 1 Instituto Nacional de Traumatologia e Ortopedia Jamil Haddad, Rio de Janeiro, RJ, Brazil

**Keywords:** chronic pain, hip, osteoarthritis, hip, pulsed radiofrequency treatment, dor crônica, osteoartrite do quadril, quadril, tratamento por radiofrequência pulsada

## Abstract

**Objective** To evaluate the role of pulsed radiofrequency (PRF) in the pain management of patients with hip osteoarthritis (OA) and surgical indication.

**Methods** We selected 30 patients from the waiting list for total hip arthroplasty, with wait time ranging from 1 to 3 years. The OA degree was measured radiographically according to the Tönnis classification. Patients underwent PRF in the surgical center, performed by two senior hip surgeons from the hospital. The procedure was fluoroscopy-guided and occurred under anesthetic sedation. One nurse assessed all patients before and after PRF using the short form-36 questionnaire.

**Results** From the initial sample of 30 patients, only 13 underwent PRF. Per the Tönnis classification, one subject was type I, four were type II, and eight were type III. The results showed an improvement in pain in 6 patients (46%), general health status in 9 (69%), social aspects in 8 (62%), and mental health in 3 (8%). Furthermore, 2 subjects (15%) reported pain worsening after PRF, and 3 (23%) reported general health status worsening.

**Conclusion** In more advanced degrees of hip joint degeneration (Tönnis III), the technique was flawed, risky, and unsatisfactory. The data obtained question PRF cost-effectiveness and its indication for patients with hip OA as a safe and effective alternative conservative treatment.

## Resumo

**Objetivo** Avaliar o papel da radiofrequência pulsátil (RFP) no manejo álgico de pacientes com osteoartrite (OA) do quadril com indicação cirúrgica.

**Métodos** Foram selecionados 30 pacientes da fila de cirurgia para artroplastia total de quadril com tempo de espera de 1 a 3 anos. O grau de OA foi mensurado radiograficamente de acordo com a classificação de Tönnis. As RFPs foram realizadas no centro cirúrgico por dois cirurgiões seniores especialistas em quadril do hospital. O procedimento foi guiado por fluoroscopia e ocorreu sob sedação anestésica. Todos os pacientes foram avaliados no pré- e pós-procedimento através do questionário Short Form-36 por uma única enfermeira.

**Resultados** Dos 30 pacientes previamente selecionados, apenas 13 realizaram a RFP. Dentre eles, um era classificado como Tönnis tipo I, quatro como II e oito como III. Os resultados evidenciaram a melhora do quadro álgico em apenas 6 pacientes (46%), do estado geral de saúde em 9 (69%), dos aspectos sociais em 8 (62%) e da saúde mental em 3 (8%). Além disso, 2 pacientes (15%) chegaram a relatar piora do quadro álgico após a RFP e 3 (23%) relataram piora do estado de saúde geral.

**Conclusão** Em graus mais avançados de degeneração articular do quadril (Tönnis III) a técnica se mostrou falha, arriscada e insatisfatória. Os dados obtidos colocam em questão não só o custo-efetividade da técnica, mas sua indicação para os pacientes com OA do quadril como tratamento conservador alternativo seguro e eficaz.

## Introduction

Populational aging and the increased physical demand in sports and work activities result in a considerable increase in hip osteoarthritis (OA) prevalence. This prevalence increases with age and, after 85 years old, one in four subjects has symptomatic hip OA. Populational studies report a range of hip OA incidence probably due to clinical and radiological dissociation. As such, chronic pain, stiffness, limited range of motion, and instability are significant issues. 1

The estimated prevalence of chronic hip pain in subjects aged over 45 is 7% in men and 10% in women. 2 The quality of life (QoL) of these patients has a direct association with pain duration and the need for prolonged searches for conservative strategies for pain relief, such as physical therapy, nonsteroidal antiinflammatory drugs, opioids, and intraarticular corticosteroid injections. These methods often provide partial and scarce symptomatic relief. 3

Several intra- and extraarticular pain sources are primary focus for hip pain, hindering their differentiation. As such, radiofrequency (RF) and intraarticular injections help to elucidate the pain source. Although the literature remains controversial, RF use has been increasing as an alternate treatment for joint pain when it is refractory to other available conservative methods, and in cases with surgical contraindication. 4 5

Anatomical models showed that the sensory anatomy innervating the hip joint consists of capsular branches of the femoral and obturator nerves, which are the major target points for RF neuromodulation guided by fluoroscopic imaging in the anteroposterior (AP) pelvis. 1

The Tönnis classification is among the best-known and most widely used worldwide for hip OA assessment. The study of a simple AP radiograph of the pelvis is enough for this classification, which initially described three progressive degrees of joint degeneration. In 1999, grade 0 was added, corresponding to subjects without the disease. Type I describes patients with mild OA, minimal joint space narrowing, increased sclerosis, and absent or minimal loss of sphericity of the femoral head. Type II demonstrates moderate OA with small cysts, moderate joint space narrowing, and moderate loss of head sphericity. Type III describes patients with advanced disease, which includes severe OA with large cysts, severe joint space narrowing, severe loss of head sphericity, and avascular necrosis. 6

The main objective of this study was to evaluate the improvement in the QoL of patients with hip OA who underwent PRF immediately, 2, 4, and 6 months after the procedure using the short form-36 (SF-36) questionnaire. The second objective was to establish a protocol for performing RF in the hospital for pain relief in patients on the waiting list for surgery.

## Materials and Methods

This observational, noncontrolled study occurred in our hospital from May to September 2022. We selected two senior hip surgeons from the same hip surgery group to conduct the study. One nurse performed the initial assessment of the QoL of all patients before the procedure, using the SF-36 questionnaire. All patients signed an informed consent form (ICF) and the Ethics Committee, affiliated with Plataforma Brasil, approved the study under opinion number 6.145.444 and CAAE number 69626023.1.0000.5273.

### Patients

We initially selected 30 patients, from both genders, with hip OA in the total hip arthroplasty (THA) waiting list. The inclusion criteria were patients over 50-years-old, presenting the primary disease in the hip, and on the waiting list from 1 to 3 years. We excluded patients under 50-years-old, with secondary hip OA, previous surgeries on the affected hip, who underwent anesthetic infiltration less than 6-months, and on the waiting list for less than 1 and more than 3 years.

### Classification

A resident physician in Orthopedics and Traumatology at the same hospital studied all hips to determine their Tönnis classification ( Table 1 ) using the MDICON imaging software.

**Table 1:** Tönnis classification

Classification	Description
0	No hip OA signs.
I	- Mild OA. - Minimal joint space narrowing and mild sclerosis. - Absent or minimal loss of head sphericity.
II	- Moderate OA. - Moderate joint space narrowing with small cysts. - Moderate loss of head sphericity.
III	- Severe OA. - Severe joint space narrowing with big cysts. - Severe head deformity.

**Abbreviation:** OA, osteoarthritis.

**Note:** Adapted from Tönnis and Heinecke 1999. 5

### Pulsed Radiofrequency

Each surgeon would perform 15 RF procedures randomly. However, we only performed 13 procedures, due to the unsatisfactory partial outcomes, as some patients developed worsening pain. Pulsed radiofrequency (PRF) uses a generator with 45 V amplitude and a duration of 2 times per second. The generator modifies parameters in real-time to reach the desired local temperature. In this method, the maximum temperature was 42° Celsius without causing irreversible tissue damage or motor fiber involvement.

### Procedure

**Fig. 1:**
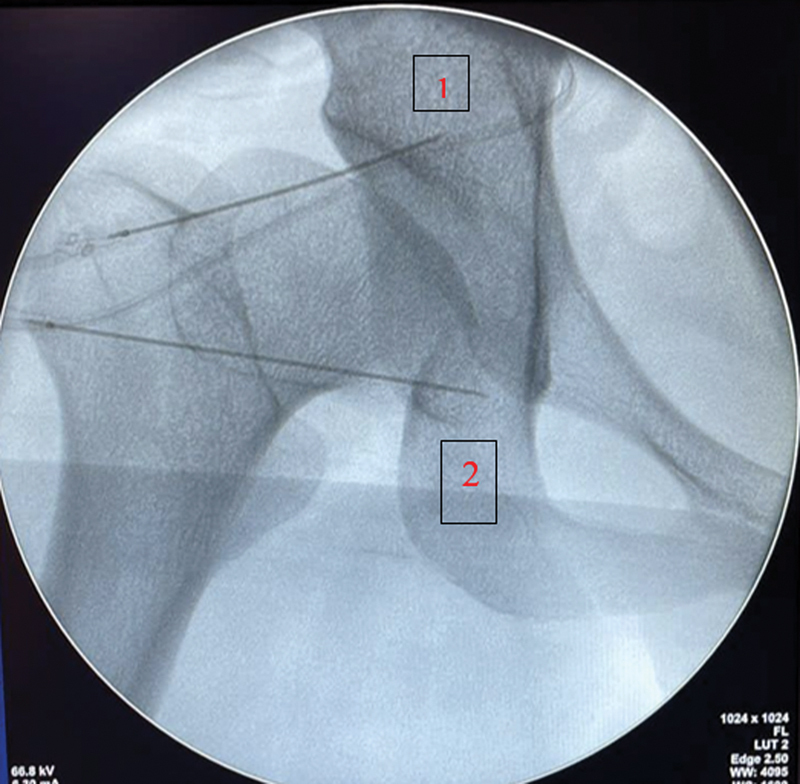
(1) Femoral nerve (2) Obturator nerve. Source: Fluoroscopic imaging from the hospital.

All patients were taken to the surgical center and underwent light sedation throughout the procedure and local anesthesia with 2% lidocaine in the affected hip. We used two PRF tips from SOLIEVO (Sollievo Medicina Especializada, São Caetano do Sul, SP, Brazil) for each subject. We inserted one tip into the sensory branches of the obturator nerve, immediately inferior to the teardrop, and one tip into the sensory branches of the femoral nerve, inferomedial to the anterior inferior iliac spine (AIIS). These anatomical parameters were obtained through an AP fluoroscopic image of the hip ( Fig. 1 ). After tip I al of methylprednisolone at 20 mg and ropivacaine 1% into each cannula and applied a compressive dressing.

### Short Form-36

The SF-36 is a widely used QoL measure developed in the 1980s in the USA. The questionnaire has 11 questions and 36 items, including 8 components (domains or dimensions) representing functional capacity (10 items), physical aspects (4 items), pain (2 items), general health status (5 items), vitality (4 items), social aspects (2 items), emotional aspects (3 items), mental health (5 items), and one question comparing patients' perception of their current health now and 1-year prior. 7 8

### Statistical Analysis

The values of each SF-36 domain were discrete and ranged from 0 to 100. Additionally, the data presented a nonnormal distribution according to the Shapiro-Wilk test. Thus, values were reported as median (interquartile range [IQR]), and the comparison between pre- and post-PRF values used the Wilcoxon signed-rank test, and the Spearman rank correlation coefficient determined associations between changes in each domain.

## Results

Our results include only 13 of the 30 previously selected patients. Per the Tönnis classification, eight subjects were type III, four were II, and one patient was type I. All SF-36 assessments pre-PRF occurred the day of or before the procedure, while for post-PRF the assessments occurred within 49 days, with the shortest and longest intervals being 25 and 96 days, respectively.

**Fig. 2:**
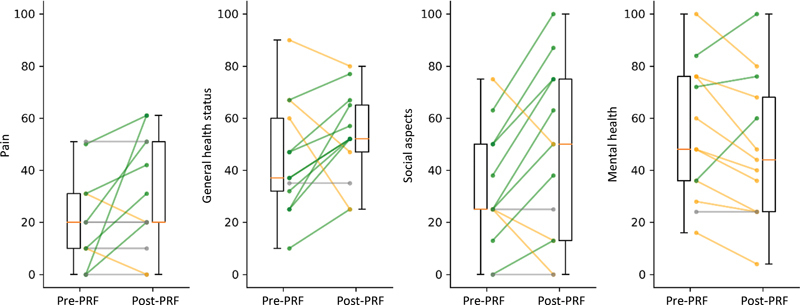
Variation in the domains assessed before and after pulsed radiofrequency. The group values are presented by boxplots and the individual values by lines. The green lines indicate improvement in the domain, orange indicate worsening, and gray indicate value maintenance. **Abbreviation:** PRF, pulsed radiofrequency.

Table 2 shows the pain, general health status, social aspects, and mental health domain values. We observed pain improvement in only 6 patients (46%), maintenance of the pre-PRF state in 5 (38%), and worsening in 2 (15%). The general health status improved in nine patients (69%), remained the same in 1 (8%), and worsened in 3 patients (23%). Social aspects improved in 8 patients (62%), remained the same in 2 (15%), and worsened in three (23%). Mental health improved in only 3 patients (23%), remained stable in 1 (8%), and worsened in 9 (69%). Fig. 2 shows the individual variation in these domains.

**Table 2:** Quality of life domains

	Pre-PRF	Post-PRF	*p-* value
**Pain**	20 (21)	20 (31)	0.057
**General health status**	37 (28)	52 (18)	0.168
**Social aspects**	25 (25)	50 (62)	0.053
**Mental health**	48 (40)	44 (44)	0.169

**Abbreviations:** PRF, pulsed radiofrequency; *p* , *p* -value of the Wilcoxon signed-rank test.

**Fig. 3:**
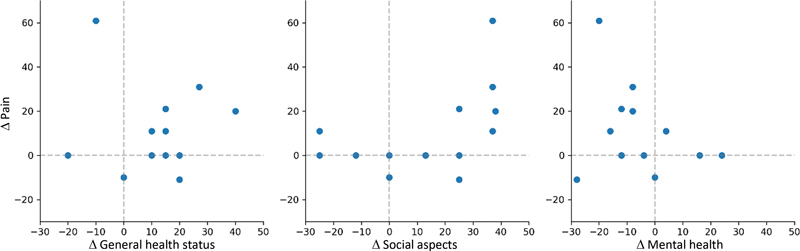
Scatter diagram of variations in the domains evaluated. Each point indicates a subject and the dotted reference lines indicate the maintenance of values before and after pulsed radiofrequency. Points located in the upper right quadrant indicate subjects with simultaneous improvement in both domains, while points located in the lower left quadrant indicate simultaneous worsening.

We did not observe any correlation between variations in the pain, general health status, and mental health domains ( Fig. 3 ). We detected a borderline value in the statistical correlation test between variations in pain and social aspects domains ( *p* = 0.056).

We interrupted the study due to the lack of partial beneficial results for patients after performing 43.3% of PRF procedures.

## Discussion

According to Giaccari et al., 10 OA is the most prevalent joint disorder in the world and one of the main causes of morbidity and functional disability. Hip OA is its second most common form. In 2019, OA prevalence in the last 10 years increased by 113.25%, going from 247.51 million affected people in 1990 to 527.81 million in 2019. Thus, the literature reported that health service costs for this condition represent 1 to 2.5% of the gross national product of developed countries. This value must increase 4-fold by 2030. 11

In the last 10 years, the waiting time for THA in our hospital from entering the list until surgery is 3.1 years. These patients with chronic pain often overuse analgesic and antiinflammatory medications and receive successive intraarticular steroid injections with limited action. Considering the current reality in our hospital, we envisioned PRF as a potential alternative and effective method for improving pain in these patients and, as a result, their QoL.

Although RF is commonly used for chronic musculoskeletal pain, 10 the literature regarding PRF in patients with hip OA is scarce. In 2017, Short et al. 1 and Bhatia et al. 3 reviewed 14 articles and demonstrated the great potential for reducing secondary pain in up to 3 years, in addition to improving walking, using RF on the sensory innervation of the hip (obturator, accessory obturator, and femoral nerves). Complications of the procedure are rare and involve vascular injury, neuritis, hematoma formation, and inadvertent ablation of motor branches of the obturator and femoral nerves.

The great advantage of PRF is the neuromodulation effects of the local electric field by altering synaptic transmission and, consequently, less damage to local tissues and less pain due to deafferentation. 12 Cooled RF (CFR) is similar to PRF but cooled with water through a probe, reaching 60°C. It can generate a neuronal lesion of a larger area, but its cost can be almost two-fold higher than PRF. 13

In another study published in 2015, 15 patients with mild-to-moderate hip OA, Tönnis types I and II, underwent PRF and were compared with 14 patients who did not undergo the procedure and received conservative treatment with paracetamol, nonsteroidal antiinflammatory drugs (NSAIDs), and opioids. Patient assessment for pain and hip function used the visual analog scale (VAS) and the Oxford hip score (OHS), and the subjects received pain medications before the procedure and at 1, 4, and 12 weeks after. The VAS and OHS scores showed a significant improvement in pain and hip function among PRF patients in all weeks evaluated. These patients also used fewer analgesics after the procedure. 14

This study had some limitations. It is known that cooled RF tips can achieve neuromodulation diameters greater than PRF tips. However, due to their high cost, they could not be used. Since 61.5% of the hips were Tönnis type III, presenting severe degeneration, we strongly believe that PRF has no function in them. Further studies with less degenerated hips would be of great value for its validation as an alternative treatment for hip OA.

## Conclusion

This study had to be interrupted with only 43.3% of the patients scheduled to undergo PRF, due to the discouraging partial outcomes. Therefore, we must question whether the investment in this technique, considered very expensive, is worthwhile and effective in improving QoL of patients with hip OA.

## References

[1] Short A J Barnett J JG Gofeld M Anatomic Study of Innervation of the Anterior Hip Capsule: Implication for Image-Guided Intervention Reg Anesth Pain Med 2018 43 02 186 192 29140962 10.1097/AAP.0000000000000701

[2] Kapural L Jolly S Mantoan J Badhey H Ptacek T Cooled Radiofrequency Neurotomy of the Articular Sensory Branches of the Obturator and Femoral Nerves - Combined Approach Using Fluoroscopy and Ultrasound Guidance: Technical Report, and Observational Study on Safety and Efficacy Pain Physician 2018 21 03 279 284 29871372

[3] Bhatia A Hoydonckx Y Peng P Cohen S P Radiofrequency Procedures to Relieve Chronic Hip Pain: An Evidence-Based Narrative Review Reg Anesth Pain Med 2018 43 01 72 83 29140960 10.1097/AAP.0000000000000694

[4] Hernández-González L Calvo C E Atkins-González D Peripheral Nerve Radiofrequency Neurotomy: Hip and Knee Joints Phys Med Rehabil Clin N Am 2018 29 01 61 71 29173665 10.1016/j.pmr.2017.08.006

[5] Diwan S Gupta A Sancheti P Sanghvi S Panchawagh S Percutaneous pulsed radiofrequency ablation of articular nerves of the hip joint in patients with chronic hip pain refractory to conventional analgesics Agri 2024 36 02 83 91 38558402 10.14744/agri.2023.90236

[6] Kovalenko B Bremjit P Fernando N Classifications in Brief: Tönnis Classification of Hip Osteoarthritis Clin Orthop Relat Res 2018 476 08 1680 1684 30020152 10.1097/01.blo.0000534679.75870.5f PMC6259761

[7] Syddall H E Martin H J Harwood R H Cooper C Aihie Sayer A The SF-36: a simple, effective measure of mobility-disability for epidemiological studies J Nutr Health Aging 2009 13 01 57 62 19151909 10.1007/s12603-009-0010-4 PMC2654814

[8] Grönkvist R Vixner L Äng B Grimby-Ekman A Measurement Error, Minimal Detectable Change, and Minimal Clinically Important Difference of the Short Form-36 Health Survey, Hospital Anxiety and Depression Scale, and Pain Numeric Rating Scale in Patients With Chronic Pain J Pain 2024 25 09 104559 38734041 10.1016/j.jpain.2024.104559

[9] Soffin E M Wu C L Regional and Multimodal Analgesia to Reduce Opioid Use After Total Joint Arthroplasty: A Narrative Review HSS J 2019 15 01 57 65 30863234 10.1007/s11420-018-9652-2 PMC6384219

[10] Giaccari L G Coppolino F Aurilio C Pulsed Radiofrequency and Platelet Rich Plasma in Degenerative Joint Arthritis: Two Case Reports and Literature Analyses Life (Basel) 2023 13 06 1334 37374117 10.3390/life13061334 PMC10302511

[11] Fan Z Yan L Liu H The prevalence of hip osteoarthritis: a systematic review and meta-analysis Arthritis Res Ther 2023 25 01 51 36991481 10.1186/s13075-023-03033-7 PMC10053484

[12] Boogaard L L Notten K Kluivers K Van der Wal S Maal T JJ Verhamme L Accuracy of augmented reality-guided needle placement for pulsed radiofrequency treatment of pudendal neuralgia: a pilot study on a phantom model PeerJ 2024 12 e17127 38560457 10.7717/peerj.17127 PMC10981882

[13] Gupta A Huettner D P Dukewich M Comparative Effectiveness Review of Cooled Versus Pulsed Radiofrequency Ablation for the Treatment of Knee Osteoarthritis: A Systematic Review Pain Physician 2017 20 03 155 171 28339430

[14] Chye C L Liang C L Lu K Chen Y W Liliang P C Pulsed radiofrequency treatment of articular branches of femoral and obturator nerves for chronic hip pain Clin Interv Aging 2015 10 569 574 25834413 10.2147/CIA.S79961 PMC4365740

